# Extracellular adenosine 5’-triphosphate concentrations changes in rat spinal cord associated with the activation of urinary bladder afferents. A microdialysis study

**DOI:** 10.1590/S1679-45082016AO3794

**Published:** 2016

**Authors:** Jeová Nina Rocha

**Affiliations:** 1 Faculdade de Medicina de Ribeirão Preto, Universidade de São Paulo, Ribeirão Preto, SP, Brazil.

**Keywords:** Adenosine triphosphate, Microdialysis, Visceral afferents, Spinal cord

## Abstract

**Objective:**

To determine adenosine 5’-triphosphate levels in the interstice of spinal cord L_6_-S_1_ segment, under basal conditions or during mechanical and chemical activation of urinary bladder afferents.

**Methods:**

A microdialysis probe was transversally implanted in the dorsal half of spinal cord L_6_-S_1_ segment in female rats. Microdialysate was collected at 15 minutes intervals during 135 minutes, in anesthetized animals. Adenosine 5’-triphosphate concentrations were determined with a bioluminescent assay. In one group of animals (n=7) microdialysate samples were obtained with an empty bladder during a 10-minutes bladder distension to 20 or 40cmH_2_O with either saline, saline with acetic acid or saline with capsaicin. In another group of animals (n=6) bladder distention was performed and the microdialysis solution contained the ectonucleotidase inhibitor ARL 67156.

**Results:**

Basal extracellular adenosine triphosphate levels were 110.9±35.34fmol/15 minutes, (mean±SEM, n=13), and bladder distention was associated with a significant increase in adenosine 5’-triphosphate levels which was not observed after bladder distention with saline solution containing capsaicin (10µM). Microdialysis with solution containing ARL 67156 (1mM) was associated with significantly higher extracellular adenosine 5’-triphosphate levels and no further increase in adenosine 5’-triphosphate was observed during bladder distension.

**Conclusion:**

Adenosine 5’-triphosphate was present in the interstice of L_6_-S_1_ spinal cord segments, was degraded by ectonucleotidase, and its concentration increased following the activation of bladder mechanosensitive but not of the chemosensitive afferents fibers. Adenosine 5’-triphosphate may originate either from the central endings of bladder mechanosensitive primary afferent neurons, or most likely from intrinsic spinal neurons, or glial cells and its release appears to be modulated by capsaicin activated bladder primary afferent or by adenosine 5’-triphosphate itself.

## RESUMO

**Objetivo:**

Determinar as concentrações extracelulares do 5’-trifosfato de adenosina no interstício dos segmentos medulares L_6_-S_1_, em condições basais ou durante a ativação mecânica e química das fibras aferentes vesicais.

**Métodos:**

Um cateter de microdiálise foi implantado no sentido transversal na parte dorsal da medula espinal, entre os segmentos L_6_-S_1_ de ratas. O microdialisado foi coletado em intervalos de 15 minutos, durante 135 minutos, com os animais anestesiados. A concentração de 5’-trifosfato de adenosina nas amostras foi determinada mediante ensaio de bioluminescência. Em um grupo de animais (n=7), as amostras de microdialisado foram obtidas com a bexiga vazia, com distensão da bexiga para volume de 20 ou 40cmH_2_O, com solução salina, solução salina com ácido acético, ou solução salina com capsaicina. Em outro grupo (n=6), foi realizada com a bexiga distendida, e a solução para microdiálise continha o inibidor de ectonucleotidase ARL 67156.

**Resultados:**

Os níveis extracelulares de trifosfato de adenosina no início do estudo foram 110,9±35,36fmol/15 minutos (média±EPM, n=13), e a distensão da bexiga causou um aumento nos níveis de 5’-trifosfato de adenosina, o que não foi observado após a distensão da bexiga com solução salina contendo capsaicina (10µM). A microdiálise com solução contendo ARL 67156 (1mM) foi associada com significante aumento dos níveis de trifosfato de adenosina extracelular, e nenhum aumento do trifosfato de adenosina foi observado durante a distensão da bexiga.

**Conclusão:**

O 5’-trifosfato de adenosina está presente no interstício do segmento L_6_-S_1_ da medula espinal, é degradado por ectonucleotidases, e sua concentração aumentou com a ativação das fibras aferentes mecanossensíveis da bexiga, mas não das quimiossensíveis. O 5’-trifosfato de adenosina pode ter sido liberado das terminações centrais dos neurônios aferentes primários mecanossensíveis ou, mais provavelmente, de neurônios espinais intrínsecos, ou ainda de células gliais. Sua liberação parece ser modulada por fibras aferentes primárias da bexiga ativadas pela capsaicina ou pelo próprio 5’-trifosfato de adenosina.

## INTRODUCTION

Adenosine 5’-triphosphate (ATP) was detected in dorsal ganglia extracts more than 50 years ago.^(1)^ A few years later, it was observed that antidromic stimulation of primary afferent fibers caused an increase in extracellular ATP levels in tissues innervated by the stimulated afferent fibers.^(2)^Since then, accumulated evidence have indicated that ATP, besides its established role as an intracellular energy source, can also act as an extra- and intercellular messenger released by a variety of cells, including neurons in the central and peripheral nervous system.^(3,4)^

Adenosine 5’-triphosphate has been also proposed to be a neurotransmitter released by the central endings of some primary afferent neurons,^(5,6)^ however, most evidence that support this proposal is indirect. For example, electrophysiological studies (patch-clamp) on spinal cord slices showed that two purported P_2_ receptor selective antagonists [pyridoxalphosphate-6-azophenyl-2´,4´-disulphonic acid (PPADS) and suramin] decreased, whereas the ectonucleotidase inhibitor (ARL 67156) increased the amplitude of lamina V neurons excitatory postsynaptic currents (EPSC), evoked by dorsal root stimulation.^(6)^ In addition, studies *in vitro*, with primary cultures of neonate rat dorsal horn neurons (laminae I-III), showed that postsynaptic currents were reduced by purinergic receptor antagonists.^(7)^ Synaptosomes prepared from spinal dorsal horns release ATP when depolarized with K.^(8)^ Capsaicin pre-treatment of the animals did not alter the amount of ATP released by synaptosomes. Since the quantity of ATP released by spinal cord dorsal horn synaptosomes was only partially reduced by dorsal rhizotomy, it was proposed that ATP originated mainly from capsaicin-insensitive primary afferents fibers (Ab e Ad), as well as from spinal cord intrinsic neurons.^(8,9)^ In addition, by using intrathecal administration of novel and selective P_2_X antagonists, Kaan et al., suggested that spinal purinergic receptors may regulate afferent signals coming from the bladder.^(10)^

Early experiments in mice lacking the expression of P_2_X_3_ receptors, as well as the use of selective P_2_X antagonists, suggested that peripheral P_2_X_1_ and P_2_X_3_ receptors, at the bladder level, are involved in the micturition reflex.^(11-14)^More recently, it has been shown that ATP is released by the urothelium to activate purinergic receptors present in the peripheral endings of bladder primary afferents.^(15)^ However, recent experiments conducted in genetically engineered mice that do not express P_2_X_2_ or P_2_X_3_ receptors showed that these receptors are not essential for the normal micturition reflex.^(16)^

Whether ATP is released at the spinal level in response to afferent impulses coming from the bladder, and whether such ATP is involved in the integration of the micturition reflex has not been directly investigated.

## OBJECTIVE

To determine adenosine 5’-triphosphate levels in the interstice of spinal cord L_6_-S_1_ segment under basal conditions or during mechanical and chemical activation of urinary bladder afferents.

## METHODS

Female rats, Sprague Dawley, weighing 230-260g, were used in these experiments. All experimental procedures were approved by the Committee of Ethics in Animal Experimentation of the *Faculdade de Medicina de Ribeirão Preto da Universidade de São Paulo*, approval number 013/2014-1.

On the day before performing microdialysis, the rats were anesthetized with isoflurane (isoflurane 3-4% for induction and 1-2% maintenance dose during the surgery) and ventilated with 100% oxygen. After hair-clipping and antisepsis using betadine solution, a dorsal median surgical incision extending from T_13_ to L_5_ vertebrae was performed. The procedure to implant the microdialysis probe was conducted as previously described.^(17)^ Briefly, using a dental drill (with a 300μm diameter steel burr), a small hole was made in both lamina of L_2_ vertebra. After that, a tubular fiber (200μm diameter) 6.0cm in length, coated with a thin layer epoxy (except for a 2mm middle region that remained uncoated constituting the effective zone for microdialysis) was introduced through these holes, in order to position it in the interstice of L_6_-S_1_ spinal cord segment boundary to dialyze the dorsal horn and the dorsal commissure. The probe was fixed with cyanoacrylate and epoxy glue and its ends were attached to polyethylene catheters (PE-20), which were exteriorized in the dorsal cervical region through a subcutaneous tunnel. The cannulae were filled with 10% heparin and sealed by electrocauterium. Immediately after the surgery, all animals were injected with ampicillin (100mg/kg, intramuscular) and placed in individual cages with food and water *ad libitum*.

### Microdialysis experiments

The animals were anesthetized with urethane (1g/kg, subcutaneously). A catheter (PE-10 Clay Adams, Nova Jersey, USA) was introduced into the right femoral vein for the continuous infusion of saline solution containing 5% glucose (5mL/minute). A polyethylene (PE-60) catheter was introduced into the bladder through the urethra, and fixed to the urethral meatus in order to monitor intravesical pressure under isovolumetric conditions.

Fluorinated ethylene propylene (FEP) cannulae previously perfused with 30% ethanol (30 to 40 minutes), and washed with Milli-Q ultrapure water, for 20 minutes, were connected to the PE-20 cannulae in order to perfuse the microdialysis probe (flow rate of 5mL/minute), using microdialysis pump (model CMA-102, CMA, Sweden), with Krebs’ solution (mM: NaCl 113; KCl 4.7; CaCl_2_ 2.5; MgSO_4_ 1.2; NaHCO_3_ 2.5; KH_2_PO_4_ 1.2 and d-glucose 11.5) saturated with 95% O_2_/5%CO_2_. The bladder was distended to 20 or 40cmH_2_O for 10 minutes with 0.9% saline or with 0.9% saline solution containing 0.1% acetic acid, or with 0.9% saline containing capsaicin (10mm).

In a group of animals (n=6), 1mM of the ectonucleotidase inhibitor 6-N, N-diethyl-D-b-g-dibromomethylene-ATP (ARL 67156) was added to the dialyzing Krebs solution, 90 minutes after initiating the microdialysis. After adding the ARL 67156, microdialysis was continued for another 135 minutes. In all experiments, after 30 to 40 minutes of equilibration period, the microdialysate was collected in 15 minutes samples (75µL), with a microfraction collector (CMA-142). Samples were kept on ice until ATP determination, which was performed immediately after finishing the microdialysis.

### Determination of adenosine 5’-triphosphate

Adenosine 5’-triphosphate concentrations in the microdialysis fractions were determined in triplicates using an ATP bioluminescent assay kit (Sigma; FL-AA, MO, USA); luminescence was measured in a luminometer (Turner, TD 20/20, Sunnyvale, CA, USA). The standard curve was constructed employing disodium ATP (Sigma) dissolved in the Krebs’ solution.

### Histology

At the end of the microdialysis procedure, the animals received an overdose of urethane and were immediately perfused, via an intracardiac needle, with 150mL ice-cold saline, followed by 300 to 350mL of 4% paraformaldehyde in phosphate buffered saline (PBS). The spinal cord was then dissected and a segment containing the lumbosacral region was post-fixed in the same fixative (4°C, 24 hours). The spinal cord segment was embedded in paraffin and 10μm transverse sections were obtained with a microtome and stained with hematoxylin-eosin to identify the position of the probe.

### Statistical analysis

Adenosine 5’-triphosphate values are expressed in percentage of control values and plotted as mean±SEM. The statistical significance was determined by the Student’s *t* test for paired samples. Statistical significance was established at p<0.05.

### Reagents and drugs

Urethane, ARL 67156, ATP, acetic acid and all the constituents of the Krebs’ solution were purchased from the Sigma Chemical Co.; capsaicin was purchased from Tocris Cookson Inc.; ampicillin was purchased from Bayer.

## RESULTS

**Figure 1:**
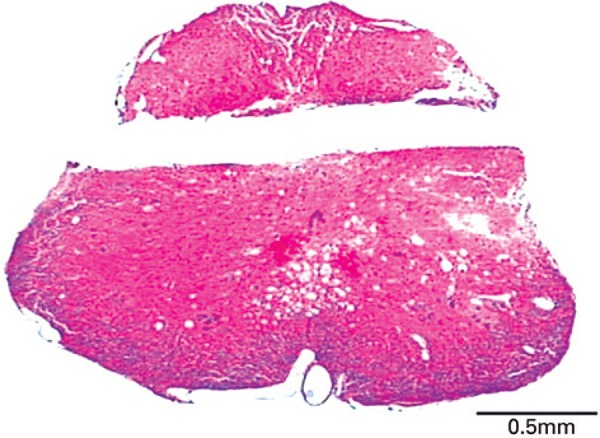
Microphotography of a representative spinal cord cross section (10µm) stained with hematoxylin-eosin showing a correctly positioned microdialysis probe. The absence of the spinal cord tissue (unstained groove) corresponds to the track where the probe was implanted

Correct probe position (100 to 150µm dorsal to the central canal) was confirmed histologically in 13 of 42 operated animals. Figure 1 shows a representative spinal cord cross section of an animal in whom the probe was considered correctly positioned. The data described and analyzed in these results correspond only to those 13 animals in which the probe was documented to be correctly positioned.

**Figure 2:**
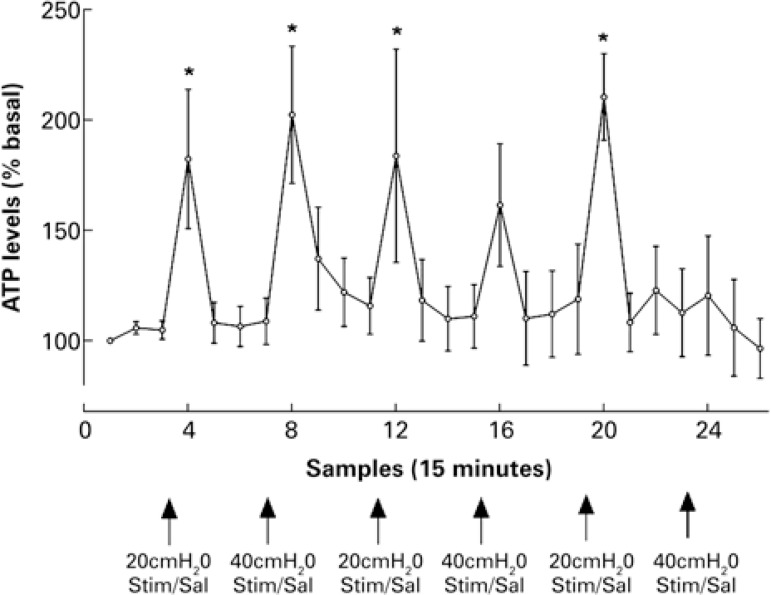
Mean±SEM adenosine 5’-triphosphate levels in successive microdialysate samples, expressed as percentage of basal values during periods of non-distended bladder, and of bladder distended to 20 or 40cmH2O with saline solution, or with saline solution containing either acetic acid (0.1%) or capsaicin 10µm, n=7; *=p<0.05 compared with preceding value

ATP: adenosine 5’-triphosphate; Stim: stimulation; Sal: saline; AA: acetic acid; Caps: capsaicin.

In the animals with the non-distended bladders (basal conditions), the amount of ATP accumulated in the microdialysate during the 15 minutes sampling period was 110.9±35.34fmol (n=13). When the bladder was distended for 10 minutes, with saline solution to about 20cmH_2_O, the amount of ATP in the microdialysate increased significantly and in a reproducible manner – this increase was transient and returned to the pre-distention values after the distension was removed (Figure 2). The increase in ATP was slightly greater, although not statistically significant, when the bladder was distended with saline solution to non-physiological pressure (40cmH_2_0) (Figure 2). Interestingly, when the bladder was distended to 20cmH_2_O with saline solution containing either acetic acid (0.1%) or capsaicin (10μm), the amount of ATP in the microdialysate was not higher than that observed when distending the urinary bladder with saline solution alone. Most intriguing, after the bladder had been distended with saline solution containing capsaicin, ATP levels in the microdialysate failed to increase during distention of the urinary bladder with saline alone (Figure 2).

**Figure 3:**
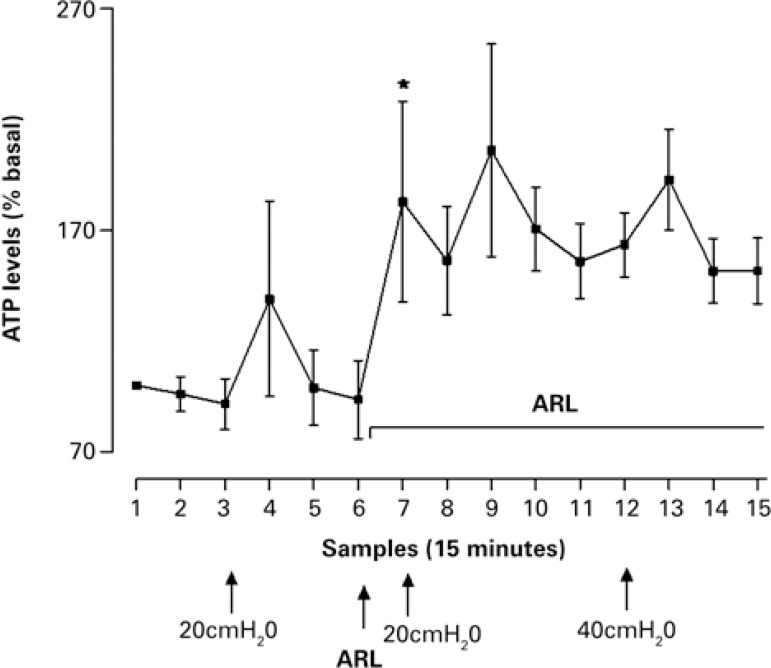
Mean±SEM adenosine 5’-triphosphate levels in successive microdialysate samples (75µL, collected in 15 minutes), expressed as percentage of basal values. Following sample 6, ARL 67156 was added to the dialyzing Krebs’ solution [(n=6) – (p<0.05)] compared with preceding value

The amount of ATP in the microdialysate increased significantly when ARL 67156 was added to the dialysing solution. ATP levels remained elevated in the continuous presence of ARL and, surprisingly, a 10-minutes bladder distension, with saline solution to either 20 or 40cmH_2_O, was not associated with any further increase of ATP in the microdialysate (Figure 3).

ATP: adenosine 5’-triphosphate.

## DISCUSSION

The results of the present study using *in vivo* spinal cord microdialysis show, for the first time, that ATP is present in the dorsal horn extracellular fluid of rat L_6_-S_1_ spinal cord segments in the absence of bladder distention, suggesting that it is released tonically in this segment of the spinal cord. Considering that the permeable portion of the probe was 2mm, the dialysated area included mainly both dorsal horns as well as the dorsal gray commissure, and given that the segment L_6_-S_1_ receives input from mechanosensitive afferent fibers innervating the urinary bladder, it is not unlikely that part of the ATP found in the extracellular fluid in the absence of bladder stimulation is released in response to inputs from “spontaneous” active bladder mechanosensitive afferent fibers described previously.^(18)^

In addition, the present results also showed for the first time that a physiological distension of the bladder (intravesical pressure of about 20cmH_2_O) is associated with an increase in extracellular ATP in the dorsal half of L_6_-S_1_ spinal segment. The observed increase is substantially less than that reported for ATP release in the brainstem chemosensitive area induced by hypercapnia,^(19)^ or in the nucleus tractus solitarii (NTS) following activation of pulmonary stretch receptors^(20)^ using microelectrode biosensors. It is likely that this increase in extracellular ATP results from its release by intrinsic spinal neurons that were activated by the central endings of bladder mechanosensitive afferents. This interpretation is consistent with data showing that dorsal horn intrinsic neurons in culture release ATP^(7)^ as well as with a recent report showing that ATP is released by intrinsic dorsal horn neurons in a vesicular nucleotide transporter (VNUT)-dependent manner.^(21)^ Alternatively, ATP could also originate from dorsal horn glial cells, since these cells can release ATP in response to glutamate.^(22)^ It is worth noting that glutamate has been proposed as the neurotransmitter released by mechanosensitive bladder primary afferents in the dorsal horn.^(23,24)^

Another important observation of this study was the fact that the increase in dorsal horn extracellular ATP, when the bladder was distended with saline solution containing acetic acid or capsaicin, well known activators of chemosensitive afferent fibers in the urinary bladder, was not greater than the one caused by the bladder distension with saline alone. This observation would indicate that the central endings of bladder chemosensitive primary neurons neither release ATP nor activate intrinsic spinal cord cells to release ATP. This interpretation is consistent with previous studies showing that the amount of ATP released by synaptosomes prepared from the dorsal horn was not diminished by the pre-treatment with capsaicin.^(8)^ These findings apparently conflict with the postulated role of ATP as a mediator in primary afferent central endings and dorsal horn neurons synapse;^(25)^ however, more recent studies have shown that ATP does not mediate fast synaptic transmission between dorsal ganglia neurons and dorsal horn neurons at spinal cord lumbar segments.^(23,26)^

Perhaps the most intriguing observation of the present study was the fact that bladder distension with saline solution, subsequent to the distension with saline solution containing capsaicin (10mm), was not associated with an increase in dorsal horn extracellular ATP levels. The simplest interpretation of this observation is that neurotransmitters released by the central terminals of capsaicin-sensitive primary afferent neurons (CSPANs) innervating the bladder, which were activated by capsaicin, exert a direct or indirect inhibitory influence on the intrinsic spinal cells that release ATP in the dorsal horn in response to inputs from mechanosensitive primary afferents.

Finally, the present findings indicate also that ATP itself could be involved in an auto-inhibitory control of its release since, after adding the ATPase inhibitor ARL 67156 to the dialyzing solution, which raised extracellular ATP levels, bladder distensions were not associated with further increases in ATP in the dorsal horn interstice. The fact that during microdialysis containing ARL 67156 ATP levels increased is consistent with the description of ectonucleotidases in the dorsal horn lumbar segments.^(27)^This auto-inhibitory control could be exerted directly on the spinal cells (neuron or glial) that release ATP, or alternatively by an indirect action on spinal interneurons evoking the release of inhibitory neurotransmitters, such as GABA and glycine as shown previously.^(28,29)^

## CONCLUSION

This study shows for the first time, using microdialysis *in vivo*, that the activation of bladder mechanosensitive afferent fibers caused an increase in extracellular adenosine 5’-triphosphate concentration in the dorsal horn of L_6_-S_1_ spinal cord segment. Whether this increase results from adenosine 5’-triphosphate released by intrinsic spinal neurons, glial cells or both, in response to synaptic input from primary afferent mechanosensitive fibers remains to be investigated. Furthermore, these findings also indicate that adenosine 5’-triphosphate release appears to be modulated by adenosine 5’-triphosphate itself and by capsaicin-sensitive primary afferent neurons activity. Finally, although further work is needed to elucidate the role of adenosine 5’-triphosphate released in the dorsal horn in modulating the micturition reflex in physiological and pathophysiological conditions, it is conceivable that spinal dorsal horn purinergic neurotransmission, activated by bladder distention, could constitute a potential pharmacotherapeutic target for clinically relevant alterations in the micturition reflex.
